# PtNiSnO_2_ Nanoframes as Advanced Electrode Modifiers for Ultrasensitive Detection of Trazodone in Complex Matrices

**DOI:** 10.3390/ijms26188861

**Published:** 2025-09-11

**Authors:** Małgorzata Suchanek, Agata Krakowska, Kamil Szmuc, Dariusz Łukowiec, Marcel Zambrzycki, Robert Piech

**Affiliations:** 1 Department of Analytical Chemistry and Biochemistry, Faculty of Materials Science and Ceramics, AGH University of Krakow, A. Mickiewicza 30 Av., 30-059 Krakow, Poland; msuchanek@agh.edu.pl (M.S.); agata.krakowska@uj.edu.pl (A.K.); 2 Department of Inorganic Chemistry and Pharmaceutical Analytics, Faculty of Pharmacy, Jagiellonian University Medical College, 9 Medyczna Street, 30-688 Krakow, Poland; 3 Faculty of Exact and Technical Sciences, Institute of Materials Engineering, University of Rzeszow, Pigonia 1 St., 35-310 Rzeszow, Poland; kszmuc@ur.edu.pl; 4 Materials Research Laboratory, Faculty of Mechanical Engineering, Silesian University of Technology, Konarskiego 18A St., 44-100 Gliwice, Poland; dariusz.lukowiec@polsl.pl; 5 Department of Biomaterials and Composites, Faculty of Materials Science and Technology, AGH University of Kraków, A. Mickiewicza 30 Av., 30-059 Krakow, Poland; zambrzycki@agh.edu.pl

**Keywords:** trazodone, platinum-nickel-doped tin oxide, EIS, voltammetry, FIA

## Abstract

A novel voltammetric sensor was constructed by modifying a glassy carbon electrode with a composite material consisting of platinum–nickel-doped tin oxide and carbon black (PtNiSnO_2_-CB/GCE), enabling highly sensitive differential pulse voltammetry (DPV) determination of trazodone HCl (TRZ). The DPV experimental parameters, including the composition of the supporting electrolyte and instrumental settings, were carefully optimized to achieve maximum analytical efficiency. Within the linear range of 1–10 µM, quantification of TRZ molecules could be performed without the preconcentration step. When applying a 60 s accumulation time (in the range 0.02–0.2 µM of TRZ), the detection limit reached 4.1 nM (1.67 mg L^−1^), indicating superior sensitivity compared to previously reported voltammetric techniques. The method demonstrated good reproducibility, with a relative standard deviation of 4.3% for 10 repeated measurements at 0.06 µM TRZ. The developed sensor exhibits excellent stability, simplicity of fabrication, and operational convenience. Its practical applicability was confirmed by the successful analysis of molecules of TRZ in diverse sample types, including pharmaceutical products, urine, plasma, river water, and artificial gastric and intestinal fluids, with recovery rates between 97.7% and 104.2%. Flow injection analysis (FIA) with amperometric detection was also performed for TRZ molecule determination.

## 1. Introduction

Trazodone HCl (TRZ), chemically known as 2-[3-[4-(*m*-chlorophenyl)-1-piperazinyl]-propyl]-1,2,4-triazolo-[4,3,a]pyridine-33(2H)-one hydrochloride, is an anti-depressant. Trazodone molecule is a serotonin reuptake inhibitor that blocks the transport of serotonin from the intercellular space back into the presynaptic neuron [1]. Additionally, it acts as an antagonist of 5-HT_2_ serotonin receptors. As a result, molecules of trazodone increase serotonin levels in the central nervous system (CNS). Serotonin is a monoamine neurotransmitter involved in the regulation of various physiological functions, including mood. According to one of the prevailing theories, reduced serotonin levels in the CNS are implicated in the pathogenesis of depression [2]. Trazodone alleviates psychomotor retardation, improves mood, exerts anxiolytic effects, and restores physiological sleep patterns. Importantly, it does not exhibit extrapyramidal side effects (i.e., it does not induce involuntary, uncoordinated movements) and does not enhance adrenergic neurotransmission. Trazodone is indicated for the treatment of depressive disorders of various etiologies, including depression accompanied by anxiety [3,4,5]. TRZ is primarily metabolized in the liver by the cytochrome P450 isoform CYP3A4. The major metabolite formed through this pathway is 3-(1-chlorophenyl)piperazine, a serotonergic agonist with a long half-life. The most common adverse effects associated with TRZ administration include nausea, insomnia, agitation, dry mouth, constipation, headache, hypotension, blurred vision, and confusion [6,7,8,9,10]. Due to these pharmacological properties and side effects, the analysis of TRZ in real-world samples, particularly in biological fluids, the gastrointestinal system, and environmental matrices, is of significant importance.

Numerous analytical techniques have been employed for the determination of TRZ molecules in both pharmaceutical formulations and biological matrices. These include capillary electrophoresis [11,12], high-performance liquid chromatography (HPLC) [13,14,15], liquid chromatography [16,17], atomic emission and absorption spectrometry [18], spectrophotometry, spectrofluorimetry [19,20,21,22], and voltammetric methods utilizing various electrode materials [23]. While chromatographic approaches offer high sensitivity and selectivity, they typically require labour-intensive and time-consuming sample preparation steps such as solid-phase extraction as well as access to advanced and costly instrumentation. Spectrophotometric techniques, although simpler, are generally inadequate for analyzing drug compounds in complex biological samples due to limited selectivity. In contrast, voltammetric methods have emerged as valuable tools for the quantitative analysis of a broad spectrum of organic and inorganic analytes, including pharmaceutical active substances, excipients, and their metabolites in biological fluids. Additionally, these electrochemical techniques provide useful insight into redox mechanisms under various experimental conditions.

The working electrode (WE) plays a pivotal role in any voltammetric system as it directly influences the analytical performance of the developed method. Various designs and electrode materials have been explored, with growing interest in solid-state electrodes. Among these, the glassy carbon electrode (GCE) and carbon paste electrode (CPE) are widely utilized due to their favourable electrochemical and mechanical properties, and, most notably, their amenability to surface modification. Functionalization of GCE and CPE surfaces has become a common strategy to enhance key analytical parameters such as sensitivity, selectivity, and detection limits. To achieve these improvements, the modifiers employed must exhibit high electrical conductivity, large surface area, electrocatalytic activity, and robust chemical and mechanical stability. A variety of materials have been successfully incorporated as modifiers, including carbon-based nanomaterials (e.g., carbon black, single- and multi-walled carbon nanotubes, graphite) [24,25,26,27,28], metal (e.g., bismuth, platinum, nickel) [29,30,31,32,33], metal oxides (e.g., TiO_2_, RuO_2_, CeO_2_) [34,35,36,37,38], and conducting polymers such as plasma-polymerized acrylonitrile [39]. More recently, the use of hybrid material combinations of two or more functional components has gained attention. An example of such an application is zirconia stabilized with yttria and admixed with neodymium oxide, titanium oxide, or platinum (YSZNd, YSZTi, Pt/YSZ) [40,41,42]. These composites synergistically integrate the advantageous properties of each constituent, often resulting in enhanced electrochemical performance. Particularly promising are hierarchical nanostructures, such as carbon nanotubes grown in situ on metal nanoparticles anchored to carbon nanofiber surfaces, offering improved surface reactivity and structural stability.

Multimetallic platinum–nickel nanoparticles are well known for their enhanced electrocatalytic activity, while simultaneously offering reduced material costs. PtNi alloys can form well-defined structures, such as octahedral and dodecahedral nanoparticles, which expose catalytically active crystallographic facets and exhibit a modified electronic structure with altered adsorption energies of electroactive species. Recent studies showed that such nanoparticles can be obtained also in the form of so-called nanoframes, which are characterized by open, three-dimensional (3D) frame-like structures composed of metals or metal oxides. Unlike solid or hollow nanoparticles, nanoframes possess a highly porous architecture with accessible edges, corners, and interior surfaces. Nanoframes have numerous applications, including energy storage [43,44], sensing [45,46], and biomedical applications [47,48]. However, the most significant application of nanoframes are in catalysis in many different reactions [49,50,51,52]. This is due to their unique morphology, which offers a large surface area, enhances mass transport, and allows reagents direct access to catalytically active sites [53,54]. For the first time, platinum–nickel-doped tin oxide has been synthesized and used for modification of a glassy carbon electrode.

This study aims to explore the analytical capabilities of a glassy carbon electrode modified with a PtNiSnO_2_-carbon black (CB) composite for the voltammetric determination of trazodone HCl (TRZ) in various matrices, including pharmaceutical formulations, urine, blood plasma, river water, and simulated gastric juice. The incorporation of carbon nanomaterials and PtNiSnO_2_ enhances the active surface area of the electrode, thereby improving its electrochemical performance. The developed sensor exhibited excellent sensitivity and low detection limits, surpassing those reported in previous voltammetric methods for molecule of TRZ analysis. Its applicability was confirmed through successful quantification of TRZ molecules in pharmaceutical samples, biological fluids, and environmental samples. Furthermore, the sensor’s performance was validated in an artificial digestive model, demonstrating its potential for detecting TRZ in complex physiological environments. These findings support the use of the proposed electrode as a versatile and reliable tool for TRZ molecule monitoring in clinical and pharmaceutical contexts. Furthermore, the proposed electrode is suitable for flow injection analysis, enabling rapid sample processing within a significantly reduced timeframe.

## 2. Results and Discussion

### 2.1. Structural and Surface Characterization of the Sensing Platform

**Figure 1:**
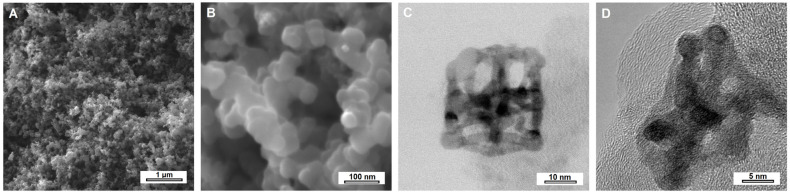
(**A**,**B**) SEM and (**C**) BFTEM and (**D**) HRTEM images of PtNiSnO_2_-CB nanohybrids.

The morphology and structure of PtNiSnO_2_-CB nanohybrids were evaluated using scanning and transmission electron microscopy, and the obtained micrographs are presented in Figure 1. The SEM images revealed the granular microstructure of carbon black with an average grain size of approx. 30 nm. The grains were relatively well separated and showed no signs of agglomeration. Observations at higher magnification using transmission electron microscopy (Figure 1C,D) confirmed the presence of PtNiSnO_2_ nanoframes on the surface of the carbon black. The nanostructures exhibited a regular shape of PtNi/SnO_2_ hollow polyhedra with distinctly defined edges, providing an increased surface area and well-exposed electrocatalytically active sites. In the HRTEM image shown in Figure 1D, a nanoframe embedded on the surface of the carbon black is clearly visible, with distinct lattice fringes of turbostratic carbon with an average interplanar spacing of d_002_ = 0.368 nm.

**Figure 2:**
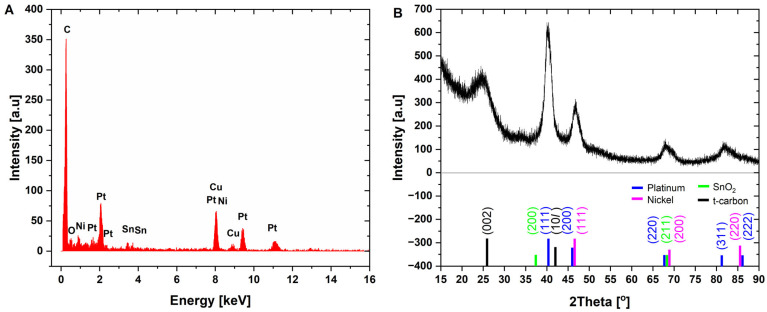
(**A**) EDX spectrum and (**B**) XRD diffraction pattern of PtNiSnO_2_-CB nanoparticles.

**Figure 3:**
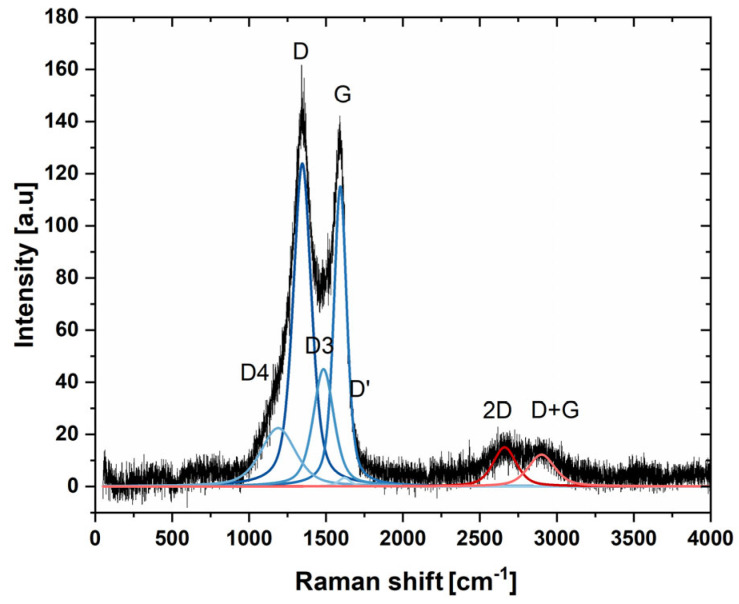
Raman spectra of PtNiSnO_2_-CB nanoparticles.

In order to evaluate the chemical composition and the structure of the materials, the X-ray diffraction patterns, EDX, and Raman spectra of nanohybrids were collected, and they are presented in Figure 2 and Figure 3. The EDX measurement taken at area of PtNiSnO_2_ nanoparticle revealed presences of carbon (46.5%), platinum (43.7%), nickel (5.9%), tin (2.5%), oxygen (1.3%), and copper, the latter originating from the sample support mesh used during measurement. The XRD diffraction pattern shown in Figure 2B was characterized by the presence of a set of peaks at 2θ of 40.5°, 47°, 68.5°, and 82°, which can be attributed to (111), (200), (220), and (311) planes of alloyed structure of PtNi [55]. Furthermore, at 25.5° and 41.0°, diffraction peaks from the (002) and (100) planes of turbostratic carbon black can be observed. No peaks from the SnO_2_ were visible due to the very small sizes of crystallites and lack of the long-range atomic order of nanoparticles. In order to provide complementary spectral information, the Raman spectrum of nanocomposite was acquired and is presented in Figure 3. The spectra consisted first- and second-order bands, characteristic of strongly disordered turbostratic carbon. The first order spectrum was deconvoluted according to Sadezky 5-band model comprising D, D’, D3, D4, and G bands [56]. The most prominent among them were the G and D bands, attributed to E_2g_ in-plane stretching of sp^2^ carbon bonds and A_1g_ breathing modes of hexagonal rings, respectively. Importantly the D band vibration mode is active only in case of disruption of transitional symmetry of hexagonal lattice, making it a reliable indicator of structural disorder in graphitic carbon materials [57]. The calculated ratio of integrated intensity of the D and G bands was 1.91 ± 0.27, indicating strongly disordered structure of carbon. The estimated in-plane crystallite size determined using Mallet-Ladeira’s methodology was L_a_ = 3.99 ± 0.42 nm [58].

### 2.2. Role and Synergistic Effect of Electrode Modifiers

**Figure 4:**
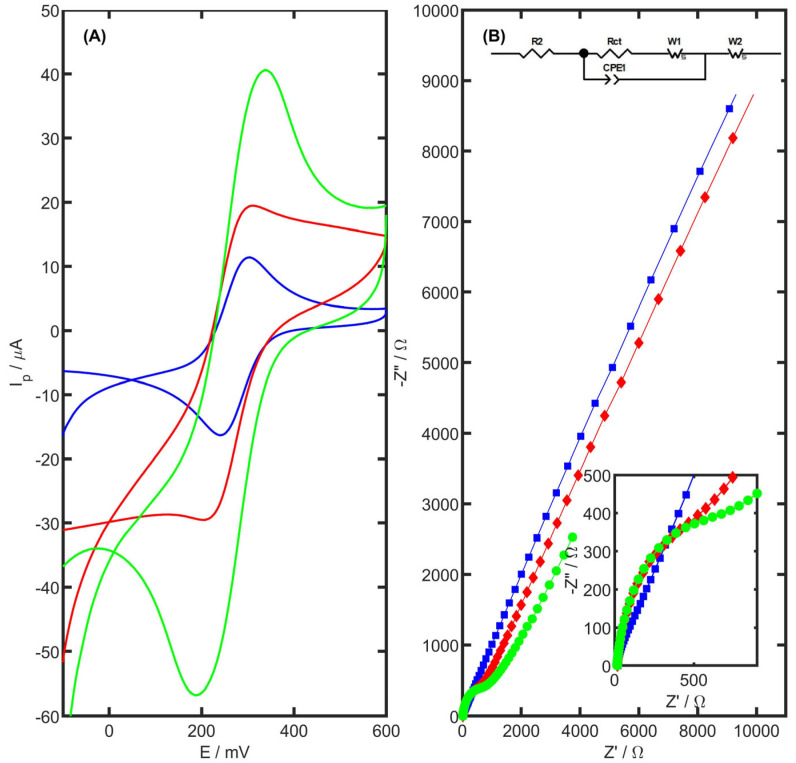
Cyclic voltammetry (**A**) and Nyquist plots (**B**) recorded for 1 mM K_3_[Fe(CN)_6_] in 1 M KCl for GC electrode (blue), CB/GC electrode (red), and PtNiSnO_2_-CB/GC (green).

Electrochemical impedance spectroscopy (EIS) and cyclic voltammetry (CV) were employed to analyze the impact of electrode surface modification on its electrochemical behaviour. Cyclic voltammograms were acquired for unmodified glassy carbon electrode, GC modified with carbon black, and GC modified with carbon black and PtNiSnO_2_ nanoparticles in a 1 mM of K_3_[Fe(CN)_6_] in 1 M KCl. The electrochemical surface area of the working electrode was calculated using the Randles–Ševčík equation [59]. The voltammograms obtained for each electrode are shown in Figure 4A, and the corresponding electrochemical parameters are presented in Table 1.

The well-defined voltammogram with symmetric anodic and cathodic peaks and a peak separation (ΔEp) of 52 mV was obtained for the bare GCE, indicating fast and reversible electron transfer. Modification with carbon black led to increased peak currents but also introduced a noticeable asymmetry between the oxidation and reduction peaks, and a broader ΔEp, suggesting perturbed charge transfer dynamics. A substantial enhancement in peak current was observed for the PtNiSnO_2_-CB/GC electrode compared to the unmodified GCE (38.78 µA vs. 13.21 µA, respectively). The redox peaks remained well-defined and nearly symmetrical, with an I_pa_/I_pc_ ratio close to unity, demonstrating improved electron transfer efficiency despite the presence of a modifier layer.

Among the modified electrodes, PtNiSnO_2_-CB/GC also exhibited the highest electroactive surface area, nearly three times greater than that of bare GCE, confirming the advantageous impact of the nanostructured composite. Although its charge transfer resistance (Rct) was higher than that of the bare GCE, it was notably lower than that of the CB/GC electrode, indicating that the incorporation of PtNiSnO_2_ nanoframes partially restores electron transfer properties compromised by the carbon black layer. The higher Rct observed for the PtNiSnO_2_-CB electrode is consistent with the increased redox peak separation for the Fe(CN)_6_^3−^/^4−^ system, indicating slower electron transfer kinetics at the modified surface. This behaviour may result from the porous carbon black layer, which, despite hindering charge transfer, facilitates mass transport. Importantly, a shift in the TRZ oxidation peak toward lower potentials was observed for the modified electrodes, suggesting enhanced electrochemical activity toward the target analyte. Taken together, these improvements in surface area, peak current, and redox symmetry point to the superior electrochemical performance of the PtNiSnO_2_-CB/GC electrode. The characteristic parameters determined based on the equation are summarized in Table 2.

Further insights into interfacial properties were obtained from EIS measurements (Figure 4B). The experiments were conducted in 1 mM K_3_[Fe(CN)_6_] with 1 M KCl, using a sinusoidal excitation signal applied over a frequency range from 100 kHz to 25 mHz. Nyquist plots were fitted to an equivalent Randles circuit (shown in the inset of Figure 4B), allowing the extraction of charge transfer resistance (*R_ct_*), Warburg impedance coefficient (*W*), double-layer capacitance (*CPE*), and effective capacitance (*C_eff_*). The capacitance was calculated using ZSimpWin 3.60 software and the following equation:

(1) $$C_{eff}={CPE}^{1/N}\times {\left[\frac{1}{R_{2}}+\frac{1}{R_{ct}}\right]}^{(N-1)/N}$$

The parameters calculated from ZSimpWin 3.60 software are summarized in Table 2.

Although the unmodified GCE exhibited the lowest charge transfer resistance (*R_ct_* = 81.7 Ω), both CB/GC and PtNiSnO_2_-CB/GC electrodes showed elevated *Rct* values (876.7 Ω and 820.4 Ω, respectively). This increase can be attributed to the formation of a porous and partially resistive surface layer due to carbon black deposition. However, the presence of PtNiSnO_2_ nanoparticles in the composite led to a measurable reduction in *R_ct_* compared to CB/GC, suggesting a partial recovery of charge transfer kinetics. It is important to note that *R_ct_* alone does not fully reflect the sensor’s performance, particularly in cases where increased surface area and catalytic activity contribute significantly to the signal enhancement. These complementary effects are further demonstrated in the following section, where differential pulse voltammetry confirms the improved sensitivity of the PtNiSnO_2_-CB modified electrode.

In addition to the classical Warburg element (*W*_1_), which reflects semi-infinite diffusion in the electrolyte, the introduction of a second Warburg-type element (*W*_2_) in the equivalent circuit significantly improved the fitting quality, particularly at lower frequencies. This additional diffusional resistance is attributed to hindered mass transport within the porous modifying layer, especially for the nanostructured composites. The marked decrease in W_2_ values across the investigated systems from 130.8 Ω for bare GC to 35.0 and 11.04 Ω for the CB and PtNiSnO_2_-modified electrodes, respectively, confirms a progressive enhancement of analyte accessibility through the surface layer. The improvement is consistent with SEM observations and BET analysis, which revealed a porous architecture enabling easier diffusion pathways. The inclusion of W_2_ provides a more realistic depiction of the overall diffusion regime at modified surfaces and reflects the structural complexity introduced by the nanomaterials. Its reduction is thus indicative of a more permeable and electrochemically active layer that facilitates both charge transfer and mass transport. Both W_1_ and W_2_ were modelled as finite-length Warburg elements (*W_s_*) in the equivalent circuit, and the reported values represent Warburg-type resistances (*R*) expressed in ohms (Ω).

### 2.3. Effect of Modifier

**Figure 5:**
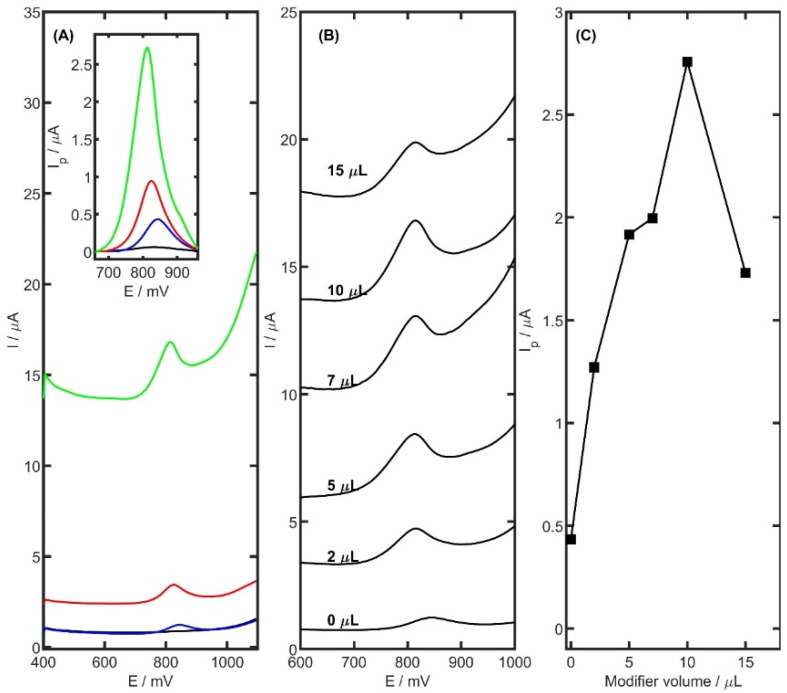
Comparison of (**A**) DP voltammograms recorded for 5 µM TRZ in 0.05 M acetate buffer at pH 5.0 (black) onto GCE (blue), CB/GCE (red), and PtNiSnO_2_-CB/GCE (green), (**B**) DP voltammograms, and (**C**) plot for difference of the volume of PtNiSnO_2_-CB modifier on the surface of GCE.

The electrochemical behaviour of TRZ molecules was further explored using differential pulse voltammetry (DPV) in acetate buffer at pH 5.0, employing three different working electrodes: unmodified glassy carbon electrode (GCE), CB-modified GCE, and PtNiSnO_2_-CB modified GCE. The comparison of the voltammetric responses obtained with these electrodes is presented in Figure 5A. As expected, the bare GCE exhibited only a slight electrochemical response toward TRZ oxidation, with a peak current of 0.43 µA. The CB/GCE showed a notably enhanced response with a well-defined oxidation peak at 825 mV and a current of 0.95 µA. The PtNiSnO_2_-CB/GCE provided the most pronounced enhancement, yielding a sharp oxidation peak at 810 mV with a current of 2.76 µA. The observed negative shift in peak potential (15 mV) for PtNiSnO_2_-CB/GCE relative to CB/GCE indicates a reduction in the activation energy for the oxidation process. This behaviour is consistent with the electrocatalytic properties of PtNiSnO_2_, which likely facilitate the electron transfer by improving surface reactivity or enhancing TRZ adsorption. Together with the previously observed increase in electroactive surface area and moderate improvement in charge transfer kinetics, these effects confirm the synergistic role of the PtNiSnO_2_-CB composite in enhancing both the sensitivity and efficiency of the voltammetric detection of TRZ.

The amount of surface modifier applied was found to significantly influence the electrochemical behaviour of the electrode. To determine the optimal volume, a series of GCEs were modified with PtNiSnO_2_-CB suspensions in volumes of 2, 5, 7, 10, and 15 µL. Electrochemical measurements were performed in the presence of 5 µM TRZ in 0.05 M acetate buffer (pH 5.0), as shown in Figure 5B,C. The greatest TRZ peak current was obtained with a 10 µL suspension, identifying it as the optimal volume for subsequent analyses. Although smaller volumes initially yielded higher peak currents, further increase to 15 µL resulted in a decline in TRZ peak current to 1.73 µA, accompanied by a noticeable rise in capacitive current at higher suspension volumes.

### 2.4. Voltammetric Profiling of TRZ Molecule: Mechanistic Insights

**Figure 6:**
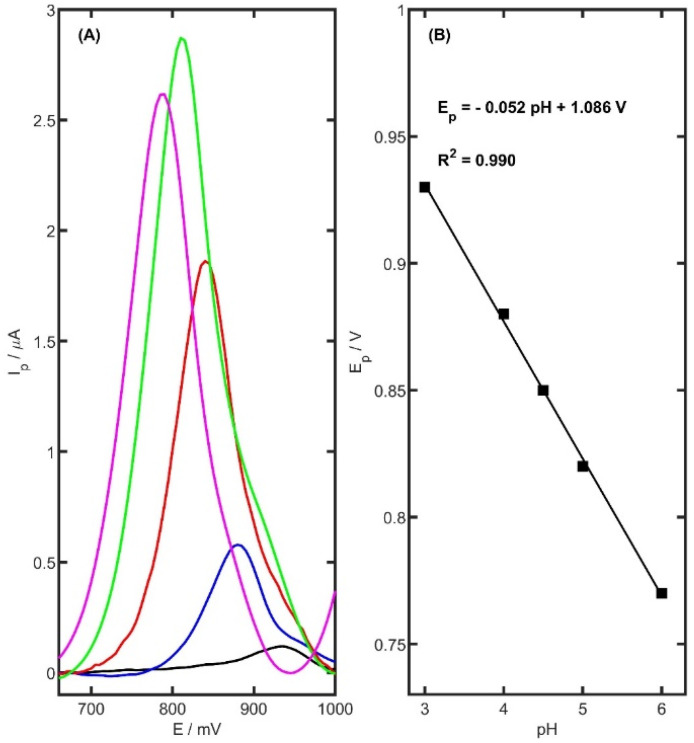
(**A**) DPV curves (after background reduction) registered for the oxidation of 5 µM TRZ in 0.05 M acetate buffer in series of various pH levels (pH 6.0—pink, pH 5.0—green, pH 4.5—red, pH 4.0—blue, pH 3.0—black). (**B**) TRZ potential peak dependence on supporting electrolyte pH in the range of 4.0–6.0.

The electrochemical response of TRZ was found to be strongly influenced by the pH of the supporting electrolyte. To determine the most effective medium for voltammetric analysis, several buffer systems were investigated. The study assessed the impact of electrolyte pH on both the position and intensity of the oxidation peak of TRZ. Five different supporting electrolytes were tested: acetate buffer (0.05, 0.1, and 0.5 M, pH 5.0), phosphate buffer (0.1 M, pH 6.0 and 7.0), potassium chloride solution (0.1 M, pH 5.0), ammonia buffer (0.1 M, pH 8.2), and borate buffer (0.05 M, pH 9.1). In contrast, oxidation peaks were observed in the following systems: 0.05 M acetate buffer (Ip = 2.71 ± 0.04 µA, Ep = 813 mV), phosphate buffer (Ip = 0.74 ± 0.03 µA, Ep = 792 mV), KCl (Ip = 0.81 ± 0.05 µA, Ep = 809 mV), ammonia buffer (Ip = 0.95 ± 0.06 µA, Ep = 773 mV), and borate buffer (Ip = 0.88 ± 0.05 µA, Ep = 740 mV). Among the tested media, the acetate buffer yielded the most pronounced anodic current. Further optimization involved evaluating the influence of ionic strength by adjusting the acetate buffer concentration (0.05, 0.1, and 0.5 M). The peak current reached a maximum at the lowest tested concentration (0.05 M). Additionally, the relationship between acetate buffer pH (ranging from 3.0 to 6.0) and TRZ oxidation current was examined. As illustrated in Figure 6, the anodic peak current increased markedly up to pH 5.0, after which a significant decline was observed.

**Figure 7:**
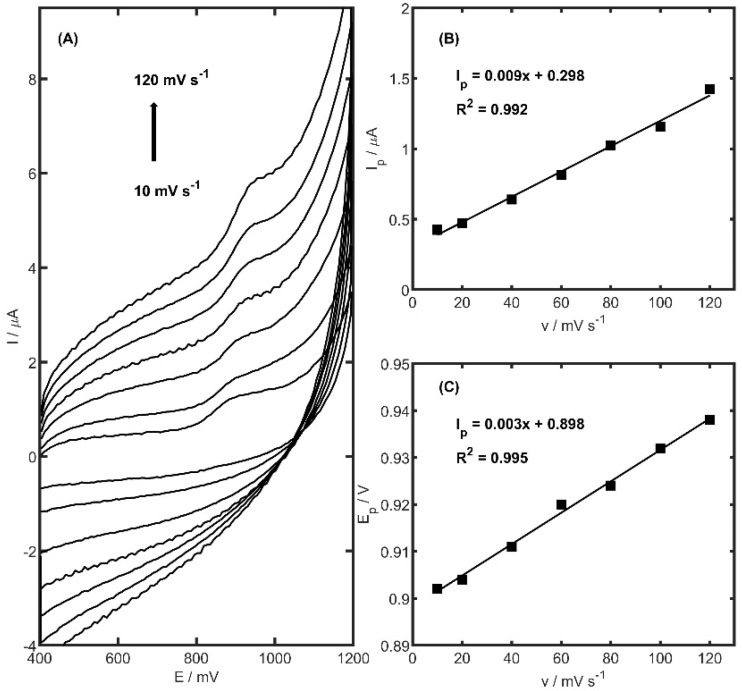
(**A**) Cyclic voltammograms of 10 µM TRZ molecule in 0.05 M acetate buffer (pH 5.0) registered on the glassy-carbon-electrode-modified PtNiSnO_2_ nanoframes and carbon black in various scan rates (10, 20, 40, 60, 80, 100, 120 mV s^−1^). (**B**) TRZ current peak and (**C**) potential peak dependence on scan rates in the range of 10–120 mV s^−1^.

Electrochemical reactions occurring at electrode surfaces are typically governed by either adsorption-controlled or diffusion-controlled mechanisms. To distinguish between these pathways, the relationship between the peak current (*I_p_*) and the scan rate (*v*) is commonly evaluated. In order to elucidate the mechanism of TRZ oxidation at the PtNiSnO_2_-CB/GCE, cyclic voltammetry was performed over a range of scan rates (10–120 mV s^−1^) within a potential window of 400–1200 mV, using a TRZ concentration of 10 µM. As illustrated in Figure 7, no cathodic peak was observed in the reverse scan, confirming the irreversible nature of the TRZ oxidation process at the modified electrode surface. Furthermore, a linear correlation between *I_p_* and *v* (Figure 7B) was identified, indicating that the oxidation of TRZ proceeds via an adsorption-controlled mechanism. Beyond the changes observed in peak current intensity, a noticeable shift in the anodic peak potential (*E_p_*) toward higher values was recorded as the scan rate increased (Figure 7C). A linear correlation between *E_p_* and the scan rate (*v*) was established, yielding a slope of 0.003 ± 0.0001, which is indicative of the kinetic nature of the electron transfer process.

The number of electrons involved in the oxidation of TRZ can be estimated using the following equation [60]:

(2) $$\alpha n=\frac{0.048}{\left|E_{p}-E_{p1/2}\right|}$$

The *αn* value calculated, where E*_p_* denotes the peak potential and E*_p1/2_* the half-peak potential, from the equation was equal to 1.04, the charge transfer coefficient (*α*) is assumed to be 0.5, and the number of electrons exchanged during the oxidation reaction to be ca. 2.

To investigate the effect of pH on the electrochemical oxidation of TRZ at the fabricated sensor, cyclic voltammetry (CV) was conducted in 0.05 M acetate buffer solutions across a pH range of 3.0 to 6.0, with a constant TRZ concentration of 5 µM. As illustrated in Figure 6B, increasing the pH induced a progressive linear shift in the anodic peak potential toward more negative values. The linear dependence of the oxidation peak potential (*E_p_*) on pH was described by the following equation:


(3) $$E_{p}=-\;0.052\;\mathrm{pH}\;+\;1.086\;V$$

The slope of the resulting linear fit (−0.052 V/pH) closely approximates the theoretical Nernstian value of −0.059 V/pH, indicating a redox process involving an equal number of protons and electrons.

**Figure 8:**
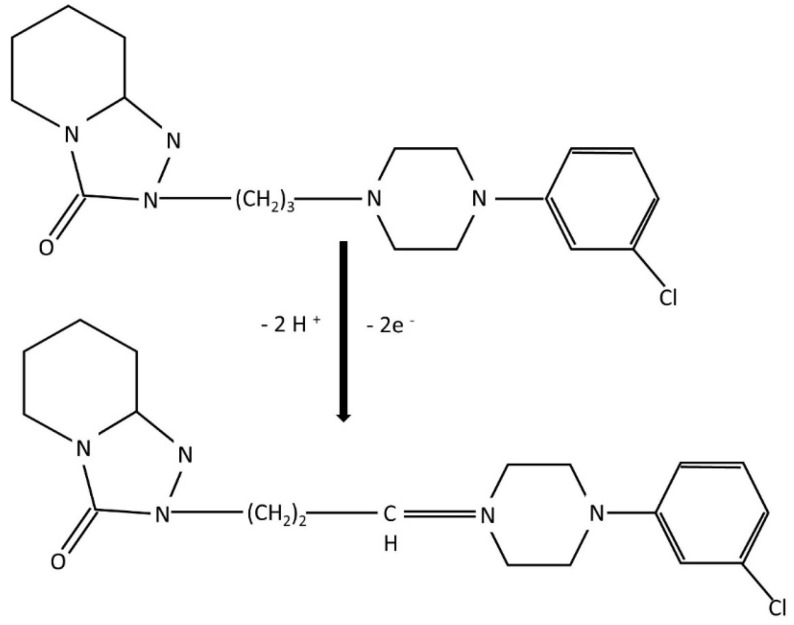
The proposed mechanism for electro-oxidation of TRZ on PtNiSnO_2_-CB/GCE.

This observation, combined with the literature reports on the electro-oxidation of amine-containing compounds, supports the hypothesis that the oxidation of TRZ primarily takes place at the piperazine moiety, which exhibits characteristic redox behaviour involving a two-electron transfer process under both acidic and basic conditions. The oxidation mechanism is proposed as follows: when the aliphatic nitrogen atom in the piperazine ring, specifically the one furthest from the aromatic benzene ring is protonated, electron transfer is initiated, accompanied by proton release (Figure 8). At pH values exceeding 8.0, the oxidation proceeds predominantly at the more basic, distal nitrogen of the piperazine ring, consistent with the established oxidative pathway for aliphatic tertiary amines. In this mechanism, TRZ first undergoes single-electron oxidation to generate a cation radical, which subsequently loses a proton and a second electron to ultimately form a quaternary Schiff base [7,61].

### 2.5. Optimization of Analytical Parameters

#### 2.5.1. DPV Method Parameters

The optimization of differential pulse voltammetry (DPV) parameters is essential for enhancing the method’s sensitivity and enabling the reliable detection of low TRZ concentrations. The optimization was performed using a 0.05 M acetate buffer at pH 5.0 with a fixed TRZ concentration of 5 µM. To maintain signal stability and reproducibility, a 15 s rest period was applied between successive voltammograms. A range of instrumental parameters was systematically investigated, including sampling time (t_s_) between 10 and 50 ms, waiting time (t_w_) from 10 to 50 ms, step potential (E_s_) in the range of 1–10 mV, and pulse amplitude (dE) varying from 5 to 100 mV in both anodic and cathodic directions. The most favourable signal response was achieved with t_s_ = t_w_ = 20 ms, E_s_ = 5 mV, and dE = 40 mV. The selected values represent a compromise between signal resolution, intensity, and background noise suppression. A pulse amplitude of 40 mV was found to significantly enhance peak current intensity without compromising signal sharpness or introducing distortion. Altogether, these parameters provided optimal electroanalytical performance for TRZ detection at the modified electrode and were subsequently employed in all further DPV measurements.

#### 2.5.2. Preconcentration Conditions

The detection of low concentrations of TRZ can be significantly improved by introducing a preconcentration step prior to the anodic scan. Both the preconcentration potential and accumulation time are key factors affecting the analytical signal in voltammetric measurements. In this study, carried out in 0.05 M acetate buffer (pH 5.0) with a TRZ concentration of 5 µM, the influence of preconcentration potential was examined within the range of 0 to 600 mV. The results demonstrated that the peak current of TRZ remained largely unaffected by changes in preconcentration potential. Consequently, a value of 400 mV was selected for further experiments.

**Figure 9:**
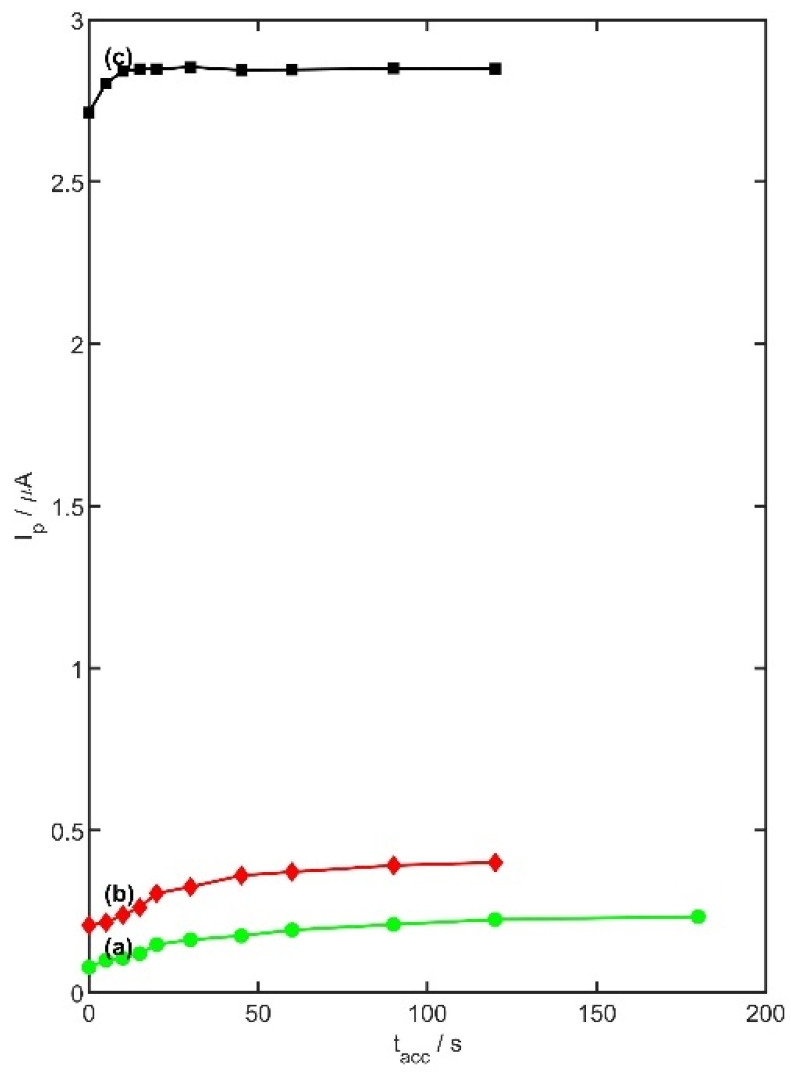
Effect of preconcentration time (0–180 s) on the peak current of trazodone at concentrations of (a) 0.05 µM, (b) 0.5 µM, and (c) 5 µM in 0.05 M acetate buffer (pH 5.0).

The effect of preconcentration time was also investigated under identical experimental conditions, with a fixed 15 s rest period between measurements. Accumulation times were varied from 0 to 180 s, and the corresponding peak current responses (*I_p_*) are presented in Figure 9. An increase in peak current was observed with longer preconcentration durations, although the maximum response was found to depend on the analyte concentration. For a 5 µM TRZ solution, the highest current (2.85 µA) was recorded at a t_acc_ of 15 s. In the case of 0.5 µM TRZ, the maximum current (0.36 µA) was observed at 45 s, while for the lowest tested concentration (0.05 µM), the peak current (0.23 µA) was achieved at 120 s. Based on these findings, preconcentration times of 0 s, 30 s, and 60 s were selected for further measurements involving TRZ concentrations of 5 µM, 0.5 µM, and 0.05 µM, respectively, using the PtNiSnO_2_CB/GC electrode.

### 2.6. Interference Study and Selectivity Evaluation

The applicability of the developed sensor for the determination of TRZ molecules (5 µM of TRZ in 0.05 M acetate buffer pH 5.0) in complex real matrices such as pharmaceutical formulations, urine, blood plasma, digestive fluids, and river water was evaluated. The influence of various organic and inorganic substances was investigated to assess potential interferences: Mg(II), Ca(II), K(I), Fe(III) (each added at a concentration of 50 µM); Cu(II), Pb(II), Zn(II), Mn(II), Al(III) (each added at a concentration of 5 µM); PO_4_^3−^, CO_3_^2−^, SO_4_^2−^, NO_3_^−^, Cl^−^ (each added at a concentration of 1 mM). The obtained results indicated that the investigated cationic and anionic species exerted no significant influence on either the peak current intensity or the oxidation potential of TRZ. Furthermore, the effect of selected organic molecules on the electrochemical response of TRZ was investigated: glucose, saccharose (each 50 µM), citric acid (100 µM added), lactose monohydrate (20 µM), ascorbic acid (20 µM), urine acid (20 µM), aspartame (20 µM), caffeine (20 µM), and acetaminophen (20 µM). In addition, the influence of selected pharmaceutical excipients on the electrochemical response of TRZ was examined: starch, talc (each 20 µM added), magnesium stearate, microcrystalline cellulose, titanium dioxide (each 5 mg per 10 mL of electrolyte), Triton X-100 (5 ppm added), humic acids (25 ppm added), saliva, gastric juice, and intestinal juice (each 20 µL per 10 mL of electrolyte). The presence of acetaminophen was the only case in which a 25% increase in the peak current was observed.

### 2.7. Analytical Study

#### 2.7.1. Calibration Graph

**Figure 10:**
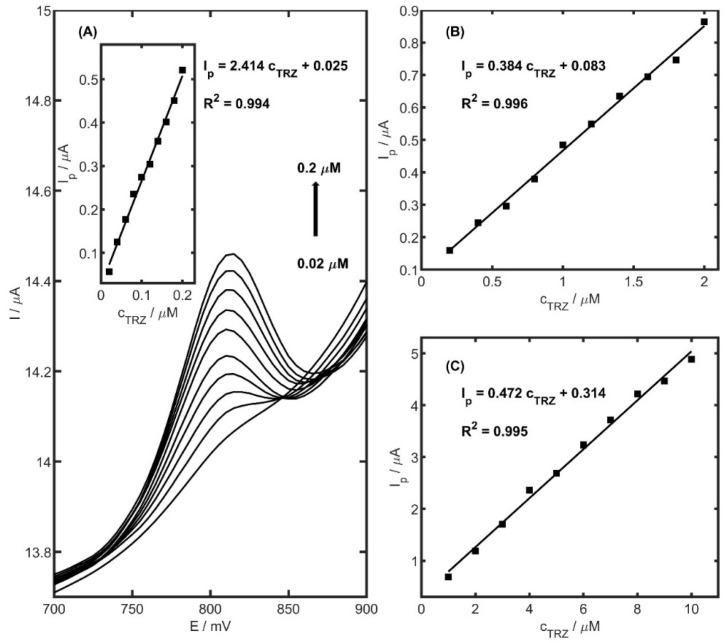
(**A**) DPV calibration voltammograms in the range of 0.02 to 0.2 µM. Inset: corresponding calibration plot. (**B**) Calibration curves obtained for TRZ concentration in the range of 0.2 to 2 µM, and (**C**) of 1 to 10 µM in 0.05 acetate buffer pH 5.0. Preconcentration time 60, 30, and 0 s, respectively.

Calibration is a fundamental step in establishing the analytical performance of the proposed method. Figure 10 presents the voltammetric calibration responses alongside the corresponding calibration curves. The experiments were performed in 0.05 M acetate buffer (pH 5.0) using an accumulation potential of 400 mV. A linear response was obtained for TRZ within the concentration range of 0.02 to 0.2 µM (Figure 10A), yielding a correlation coefficient (R^2^) of 0.994, a slope of 2.414 ± 0.076 µA µM^−1^, and an intercept of 0.043 ± 0.010 µA. The limit of detection (LOD), calculated using the standard formula LOD = 3.3 × SD/b (where SD is the standard deviation of the blank and b is the slope of the calibration curve), was found to be 4.1 nM with a preconcentration time of 60 s. This sensitivity compares favourably with previously reported electrochemical methods, as summarized in Table 3. Calibration curves and parameters for extended concentration ranges (0.2–2 µM and 1–10 µM) are provided in Figure 10B,C. In the concentration range 0.2–2 µM and the preconcentration time 30 s, a correlation coefficient (R^2^) was 0.996, a slope of 0.384 ± 0.008 µA µM^−1^, and an intercept of 0.083 ± 0.011 µA. Additionally, in the range 1–10 µM and without preconcentration time, a correlation coefficient (R^2^) equal to 0.995, a slope of 0.472 ± 0.012 µA µM^−1^, and an intercept of 0.314 ± 0.039 µA was obtained. The limits of detection for the two calibrations were calculated to be 43.0 (accumulation time of 30 s) and 130.1 nM (accumulation time of 0 s), respectively. The limit of quantification (LOQ), estimated as 10 × SD/b, was determined to be 16.7, 69.9, and 211.7 nM under the same accumulation conditions (for the range 0.02–0.2 µM, 0.2–2 µM, and 1–10 µM, respectively). The reproducibility of the electrode response was evaluated at two TRZ concentrations: 0.06 µM and 5 µM. The relative standard deviation (RSD) values were 4.3% and 3.6% (*n* = 10), respectively, confirming good repeatability. Additionally, the electrode demonstrated long-term operational stability, maintaining consistent performance over 300 successive measurements and remaining effective for at least two months under storage.

#### 2.7.2. The Application of the Procedure in Real Sample

**Figure 11:**
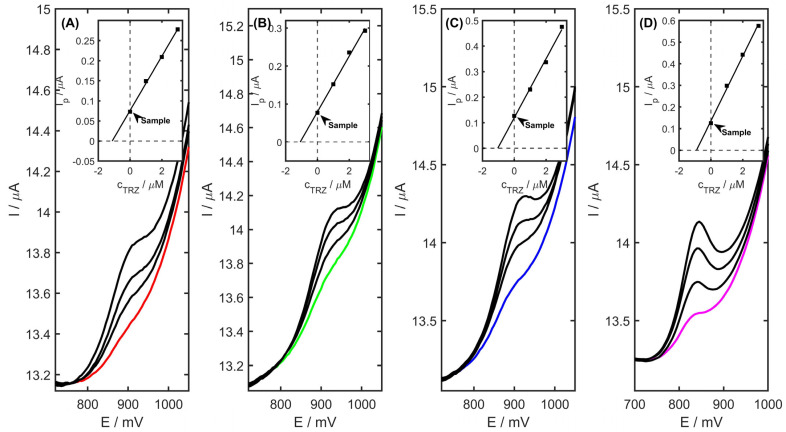
DP voltammetric responses obtained for TRZ in (**A**) tablet (red), (**B**) urine (green), (**C**) plasma (blue), and (**D**) river water samples (river water curve marked as purple, three consecutive additions of the standard marked as black). Inset: corresponding calibration plots.

The developed electrode architecture and analytical protocol were employed for the quantification of TRZ in pharmaceutical formulations, urine, plasma, and river water samples. A commercially available tablet containing TRZ was selected for analysis, with the sample preparation procedure described in detail in Section 3.3. Quantification was carried out using the standard addition method, following the protocol outlined in Section 3.5. As shown in Table 4, the amount of TRZ per tablet was determined to be 152.5 ± 2.7 mg. The obtained recovery values, ranging from 97.7% to 104.2%, confirm the reliability and applicability of the PtNiSnO_2_/CB-modified glassy carbon electrode for TRZ detection across diverse matrices, such as tablet, urine, plasma, and river water samples. The differential pulse voltammograms recorded for a spiked tablet, urine, plasma, and river water sample are depicted in Figure 11.

### 2.8. Extraction into Artificial Digestive Juices

To demonstrate the practical applicability of the proposed method, an additional experiment was performed to assess the extraction of TRZ into simulated digestive fluids. A glassy carbon electrode modified with PtNiSnO_2_ nanoparticles and carbon black was employed for the voltammetric detection of the active compound in artificial gastric and intestinal juices. For this purpose, one slow-release pharmaceutical tablet (containing 150 mg of trazodone) was dissolved in the respective media, and the resulting solutions were analyzed using the standard addition method, as described in Section 3.3.4. The experiment was carried out in triplicate. The extraction efficiencies of TRZ into artificial gastric and intestinal fluids are summarized in Table 5.

The highest extraction efficiency of TRZ in simulated digestive fluids was observed after 60 min of drug release into simulated gastric juice, as determined using the dissolution method described in the Polish Pharmacopoeia. The results indicate a slow and partial release of trazodone under acidic conditions, which is characteristic of matrix-based controlled-release systems. Trittico CR employs a hydrophilic polymer matrix that undergoes gradual hydration and swelling in the gastric environment. Prolonged exposure of the dosage form to gastric juice leads to enhanced drug release efficiency. In contrast, shorter exposure of trazodone to the gastric phase results in increased extraction efficiency in simulated intestinal fluid. The application of the Polish Pharmacopoeia dissolution protocol provides a standardized and reproducible simulation of gastrointestinal conditions, allowing for reliable comparison of release profiles across media with different pH values. In the literature, biphasic drug release from HPMC (hydroxypropyl methylcellulose)-based matrix tablets has been described, where initial swelling and limited erosion in acidic media are followed by an accelerated release phase under intestinal conditions [63,64].

### 2.9. Flow Injection System Coupled with Amperometric Detection

**Figure 12:**
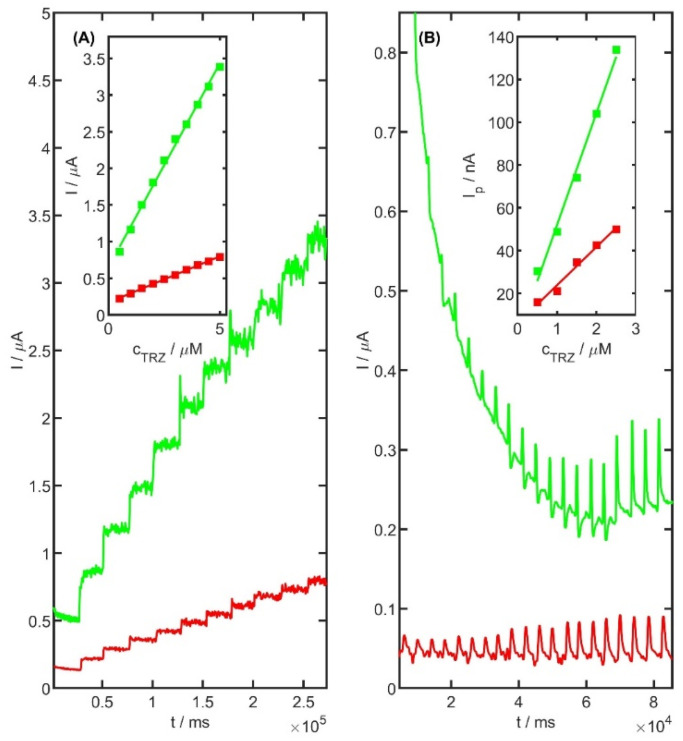
(**A**) Amperometric response on unmodified glassy carbon electrode (red line) and PtNiSnO_2_-CB modified GC electrode (green line) for TRZ concentration in the range from 0.5 to 3.5 µM. Inset: corresponding calibration curves. (**B**) TRZ calibration charts for concentration range from 0.5 to 2.5 µM using unmodified screen-printed carbon electrode (red) and 1 µL of PtNiSnO_2_-CB modified SPC electrode (green) with amperometric parameters of measurements under flow injection conditions. Inset: corresponding calibration curves.

Amperometric detection of TRZ was also carried out using the unmodified and PtNiSnO_2_ nanoframes-modified electrode. The working potential was optimized and set at 0.95 V. To ensure proper homogenization and solution stability prior to each TRZ addition, a 25 s equilibration period was applied, during which the solution was stirred at approximately 500 rpm. A linear relationship between current response and TRZ concentration was observed in the range of 0.5 to 5 μM (in 0.05 M acetate buffer pH 5.0), as shown in Figure 12A. The obtained slope of the regression line for the unmodified electrode and modified electrode was 0.129 ± 0.002 µA µM^−1^, intercept 0.157 ± 0.006 µA, r = 0.999, and 0.557 ± 0.012 µA µM^−1^, 0.651 ± 0.037 µA, r = 0.998, respectively. These findings indicate the potential applicability of the method under flow injection conditions. To verify this assumption, flow injection analysis (FIA) was subsequently employed using the PtNiSnO_2_-CB electrode as the working electrode.

To validate this approach, flow injection analysis (FIA) was employed for the determination of TRZ using the unmodified and PtNiSnO_2_ nanoframes-modified SPCE (screen-printed carbon electrode) as the working electrode. To optimize the analytical conditions, a range of working potentials and flow rates was systematically evaluated. Based on the experimental data, the optimal parameters were established as a working potential of 1000 mV and a flow rate of 2.0 mL min^−1^. The repeatability of the amperometric measurements, expressed as the relative standard deviation (RSD) for a 1 μM TRZ solution, was found to be 4.1% (*n* = 10). Figure 12B presents the calibration plots for TRZ over the concentration range of 1 to 2.5 μM. The obtained slope of the regression line for the unmodified electrode and modified electrode were slope 17.88 ± 1.33 nA µM^−1^, intercept 5.97 ± 2.20 nA, r = 0.992, slope 52.38 ± 2.79 nA µM^−1^, intercept −0.43 ± 0.06 nA, r = 0.996, respectively. To the best of our knowledge, this study reports for the first time the use of a PtNiSnO_2_-modified glassy carbon electrode for the quantification of TRZ molecules under flow injection conditions.

## 3. Materials and Methods

### 3.1. Reagents, Materials, and Instrumentation

Electrochemical measurements were carried out using a voltammetric setup designed for the analysis of biological matrices. This system incorporated a voltammetric analyser (M161) in combination with an M164 electrode stand (both from mtm-anko, Kraków, Poland), supported by a computer-controlled data acquisition system running EAQt software (http://home.agh.edu.pl/~kca/home/, accessed on 1 January 2025). A three-electrode configuration was employed, consisting of a platinum rod as the counter electrode, a double-junction Ag/AgCl/KCl reference electrode with ceramic frit, and a 3 mm diameter glassy carbon working electrode (Mineral, Łomianki-Sadowa, Poland).

The working electrode surface was modified by drop-casting a hybrid dispersion composed of carbon black (CB) and platinum–nickel-doped tin oxide (PtNiSnO_2_). All voltammetric profiles were visualized using MATLAB 2024a. Cyclic voltammetry (CV) and electrochemical impedance spectroscopy (EIS) experiments were conducted using a VersaSTAT4 potentiostat/galvanostat (Ametek Inc., Berwyn, PA, USA). All data were analyzed using the ZSimWin 3.60 software. For pH adjustment and monitoring, a CX-705 multiparameter meter (Elmetron, Zabrze, Poland) was utilized.

The flow injection analysis (FIA) system was constructed with a 50 mL sample reservoir and controlled by an 800 Dosino dosing unit paired with a 900 Touch control interface (Metrohm, Herisau, Switzerland). Injections were performed using a Rheodyne Model 7010 rotary valve, while detection was achieved via a wall-jet amperometric flow-through cell. Additionally, a thin-layer electrochemical detector equipped with screen-printed carbon electrodes (SPCEs) was employed.

The microstructure of the obtained PtNiSnO_2_-CB was evaluated using an Apreo 2 scanning electron microscope (SEM) equipped with a T2 upper in-lens secondary electron detector. Images were acquired at an accelerating voltage of 5 kV. Transmission electron microscopy (TEM) imaging was performed at Titan 80–300 (FEI) Scanning Transmission Electron Microscope (S/TEM) using an accelerating voltage of 300 kV in bright-field, dark-field, and high-resolution modes. The elemental composition of the samples was analyzed using an energy-dispersive X-ray spectroscope (EDX) coupled with the TEM (EDAX). X-ray diffraction measurements were carried out with a PANalytical X’Pert Pro diffractometer (Malvern Panalytical Ltd., Almelo, The Netherlands) equipped with a copper anode source (Cu Kα radiation, λ = 0.154 nm). Data were collected in the 2θ range of 10° to 90°, using a step size of 0.02°. The diffraction patterns were analyzed and processed using X’Pert HighScore Plus 2.0 and Fityk 0.9.8 software. Raman spectra of the materials were collected using a Horiba LabRAM HR spectrometer (Horiba Scientific, Palaiseau, France) equipped with a laser excitation source (λ = 532 nm) and an Olympus Plan M NA0.9 objective lens. Spectra were recorded in the range of 50–4000 cm^−1^ with a spectral resolution of 0.39 cm^−1^. The integration time was set to 10 s, and four individual spectra were acquired at different sample locations to obtain precise and averaged structural information. The obtained spectra were analyzed using Fittyk 0.9.8 software. For spectral fitting and deconvolution, the Pseudo-Voigt function was applied.

All reagents were used with high purity and were prepared according to the manufacturer’s protocols. All chemical reagents used were of analytical grade and were handled in accordance with the manufacturers’ protocols. Trazodone hydrochloride (TRZ) stock solution (0.01 M) was obtained from Sigma-Aldrich (St. Louis, MO, USA) and prepared in freshly double-distilled water. To minimize degradation, the solution was protected from light. Diluted working standards (0.001 M and 0.0001 M) were prepared daily using ultrapure water. Chloroplatinic acid hexahydrate (H_2_PtCl_6_·6H_2_O), nickel (II) nitrate hexahydrate (Ni(NO_3_)_2_·6H_2_O), oleylamine and tin (IV) chloride pentahydrate (SnCl_4_·5H_2_O) were purchased from Sigma-Aldrich. Acetic acid was purchased from Merck Millipore. Citric acid monohydrate, isopropanol, chloroform, and ethanol were purchased from Avantor Performance Materials (Radnor, PA, USA). Vulcan XC-72R was purchased from Cabot (Boston, MA, USA). Freeze-dried synthetic urine samples were purchased from Medichem (Steinenbronn, Germany), and human plasma was sourced from Biowest (Nuaillé, France). The substances selected for interference testing reflected compounds commonly found in biological, pharmaceutical, and environmental matrices.

### 3.2. Synthesis and Physicochemical Characterization of PtNiSnO_2_-CB Nanoframes

The solid PtNi rhombic dodecahedral nanoparticles were synthesized using a modified procedure reported by Chen et al. [53]. Briefly, a 1 mL aqueous solution containing 50 mg H_2_PtCl_6_·6H_2_O and 43 mg Ni(NO_3_)_2_·6H_2_O was injected into a mixture of 15 mL oleylamine and 10 mL oleic acid at 160 °C under an argon atmosphere. The reaction mixture was then heated to 270 °C and maintained at this temperature for 3 min after a colour change to black was observed. After cooling, the resulting suspension was subjected to centrifugation at 3000 rpm, and the collected nanopolyhedra were redispersed in 2 mL chloroform. The resulting suspension, containing solid PtNi rhombic dodecahedra, was combined with 20 mL acetic acid. After sonication for 30 min, the mixture was heated at 100 °C for 5 h under continuous stirring in air. Finally, the suspension containing PtNi nanoframes was centrifuged at 3000 rpm and subsequently washed with a hexane/ethanol mixture.

The PtNi/SnO_2_ heteroaggregates were obtained by direct synthesis of SnO_2_ nanoparticles on the PtNi nanoframes according to the following procedure. First, PtNi nanoframes were transferred into mixture of 10 mL of 0.1 M SnCl_4_·5H_2_O and 10 mL of 0.1 M citric acid solutions. The obtained solution was stirred for 30 min, then heated in a microwave oven for 12.5 min, with a maximum power of 280 W. The yielded PtNi/SnO_2_ heteroaggregates were centrifuged and washed three times with ethanol.

The obtained PtNi/SnO_2_ heteroaggregates were further deposited on Vulcan XC-72R carbon black according to the following procedure. First, the PtNi/SnO_2_-containing solutions were added dropwise to the carbon suspension and were stirred overnight. The obtained PtNiSnO_2_-CB composite was centrifuged (3000 rpm, 30 min) and then dried in air at 200 °C for 14 h.

### 3.3. Real Sample Collection and Pretreatment

#### 3.3.1. Tablet Samples

A pharmaceutical product containing 150 mg of trazodone per tablet was purchased from a local pharmacy and prepared according to a standard procedure for tablet sample preparation. Three tablets were weighed, dissolved in the supporting electrolyte, and filtered through a 0.45 µm syringe filter. The filtrate was subsequently diluted and subjected to voltammetric analysis.

#### 3.3.2. Biological Samples

The urine and plasma samples were prepared according to the manufacturer’s guidelines and previously reported procedures [40]. Commercial freeze-dried urine was reconstituted with double-distilled water, filtered through RC syringe filters (0.45 µm), and subsequently used for trazodone determination. Plasma samples were stored at −20 °C under light-protected conditions. To remove interfering proteins, 800 µL of plasma was mixed with 200 µL of 10% trichloroacetic acid (TCA), vortexed for 2 min, centrifuged at 10,000 rpm, and the supernatant was filtered (0.45 µm) prior to analysis [35,65,66].

#### 3.3.3. Surface Water Samples

A surface water sample was collected from an area affected by zinc and lead ore mining activities. Water samples were taken from a depth of approximately 30 cm, avoiding bottom sediments and visible contaminants, using pre-cleaned polyethylene containers. The sample was stored in the dark at 4 °C in a refrigerator, without the addition of any preservatives.

#### 3.3.4. The Extraction of TRZ into Artificial Digestive Juice

The release of the active pharmaceutical ingredient from the tablet into the gastrointestinal tract was also investigated. The analyzed Trittico CR tablets (1 tablet = 150 mg) were extracted into gastric and intestinal juices. To initiate the process, 4 mL of artificial saliva (prepared following Arvidson’s model) was applied to moisten the tablets. Then, 20 mL of artificial gastric juice was added to samples. The gastric juice was prepared according to the Polish Pharmacopoeia as a composition of NaCl, HCl, and pepsin. The tablet samples were maintained in artificial gastric juice for 30 and 60 min in a water bath shaker, with the temperature controlled at 36 ± 2 °C. Subsequently, the tablet was separated and pre-digested and filtrate was filtered through membrane filters. To the tablet, 20 mL of artificial intestinal juice was added and incubated for 120 min. All the above steps were conducted in three independent replicates. For analysis, the filtrates obtained from digestive juice samples were used.

### 3.4. Fabrication and Modification of Electrodes

**Figure 13:**
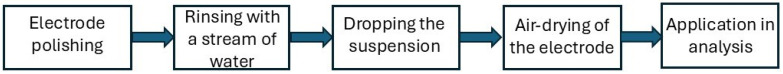
A scheme presenting the preparation and modification of electrode.

Subsequently, the GC electrode was modified by applying the synthesized PtNiSnO_2_/CB nanoparticles. Prior to the modification, the GCE surface was mechanically polished using a 0.3 µm Al_2_O_3_ suspension, followed by thorough rinsing with a stream of double-distilled water. After the cleaning step, 10 µL of the prepared composite suspension was drop-cast onto the polished surface. The modified electrode was left to dry at room temperature for 8 h under a glass cover. The resulting electrode maintained its stability and functionality for up to two months. The scheme for the modification of the electrode is presented in Figure 13.

### 3.5. Electrochemical Measurement Protocols

Differential pulse voltammetry (DPV) was employed for the quantitative determination of TRZ. The appropriate quantities of the TRZ standard solution were introduced into a 20 mL voltammetric cell, followed by dilution with acetate buffer (0.05 M, pH 5.0) to a final volume of 10 mL. During the preconcentration stage, TRZ was accumulated onto the electrode surface under magnetic stirring at approximately 500 rpm. To investigate the electrochemical characteristics of the PtNiSnO_2_-CB/GCE sensor, cyclic voltammetry and electrical impedance spectroscopy measurements (EIS) were performed. The DPV parameters were carefully optimized and set as follows: sampling time and waiting time t_p_ = t_w_ = 20 ms, step potential E_s_ = 5 mV, and pulse amplitude dE = 40 mV. Voltammograms were recorded within the potential window of 400–1100 mV. To ensure high repeatability of the MET signal, a rest period of 15 s was maintained between successive measurements.

## 4. Conclusions

In this study, a novel and highly sensitive voltammetric method was developed for the determination of TRZ molecules. For the first time, a glassy carbon electrode (GCE) was modified with platinum–nickel-doped tin oxide (PtNiSnO_2_) nanoframes in combination with carbon black (CB) to fabricate an innovative electrochemical biosensor. The proposed sensor is characterized by a straightforward fabrication procedure, low cost, and excellent reproducibility. The electrode modification significantly improved the analytical sensitivity by approximately threefold compared to a CB-modified GCE and sixfold relative to an unmodified GCE. Experimental conditions, including the composition of the supporting electrolyte and instrumental parameters, were thoroughly optimized to enhance the sensor’s performance. The influence of potential interfering compounds on the TRZ molecule signal was also systematically evaluated. Under optimal conditions, the sensor achieved a limit of detection (LOD) of 4.1 nM and a limit of quantification (LOQ) of 16.7 nM, using a preconcentration time of 60 s. These analytical figures of merit surpass those reported for previously developed electrochemical sensors targeting molecules of TRZ. The applicability of the sensor was validated through successful analysis of TRZ in pharmaceutical formulations, human urine, plasma, and environmental samples such as river water. Recovery values ranged from 97.7% to 104.2%, confirming the method’s accuracy and robustness for real sample analysis. Additionally, the extraction behaviour of TRZ in artificial gastric and intestinal fluids was examined, demonstrating the influence of extraction time on the drug’s release into the intestinal medium. The sensor enabled reliable detection of TRZ in both simulated gastric and intestinal environments. Electrochemical impedance spectroscopy confirmed that, despite the lowest charge transfer resistance being observed for bare GC, the PtNiSnO_2_-CB modification significantly improved the overall electrochemical performance by enhancing mass transport and enabling effective charge transfer through the porous sensing layer. Furthermore, the PtNiSnO_2_-CB nanoframes-modified sensor was integrated into a flow injection amperometric system. Under flow conditions, the signal for TRZ recorded using a modified screen-printed carbon electrode was approximately 2.5 times higher than that obtained with an unmodified counterpart, indicating the suitability of the proposed modification for dynamic measurement systems. This approach also offers substantial advantages in terms of reduced analysis time and operational cost.

In conclusion, the developed voltammetric platform based on nanoparticles of PtNiSnO_2_-CB-modified GCE exhibits excellent analytical performance and versatility. Its successful application to pharmaceutical, biological, environmental, and simulated digestive matrices highlights its strong potential for routine TRZ molecules analysis under both stationary and flow conditions.

## Figures and Tables

**Table 1:** The electrochemical parameters obtained for the GC electrode and its modifications of carbon black and PtNiSnO_2_ nanoframes with carbon black. Scan rate of 100 mV s^−1^.

	E_pa_, mV	E_pc_, mV	ΔE_p_, mV	I_pa_, µA	I_pc_, µA	I_pa_/I_pc_	A, cm^2^
GC	298	246	52	13.21	12.49	1.06	0.056
CB/GC	288	230	58	17.65	14.20	1.24	0.075
PtNiSnO_2_-CB/GC	320	207	113	38.79	37.77	1.03	0.165

**Table 2:** The comparison of the parameters and charge transfer resistance value derived from EIS analysis for the unmodified and modified GC electrodes.

	GC	CB/GC	PtNiSnO_2_-CB/GC
CPE [µF]	5.22	7.42	14.96
R_2_ [Ω]	18.5	16.8	15.8
R_ct_ [Ω]	81.7	876.7	820.4
W_1_ [kΩ]	152.8	36.2	0.05
W_2_ [Ω]	130.8	35.0	11.04
C_eff_ [µF]	1.46	1.14	3.92

**Table 3:** Comparison of different developed sensors used in determination of TRZ molecule.

Electrode	Technique	Electrolytic Solution	Linear Range, µM	LOD, nM	Reference
MWCNTs/GCE	DPV	Phosphate buffer pH 7.0	0.2–10	24.0	[7]
GCE	SWAAdSV	BR buffer pH 6.0	0.12–1.25	41.8	[61]
HMDE	SWCAdSV	BR buffer pH 10.0	0.008–0.61	4.32	[61]
Stationary Pt Electrode	DP	KCl and acetate buffer pH 5.5	10–50	$2.5\cdot 10^{3}$	[62]
Rotatin platinum electrode	DP	KCl and acetate buffer pH 5.5	10–50	$1.7\cdot 10^{3}$	[62]
PtNiSnO_2_-CB/GCE	DPV	Acetate buffer pH 5.0	0.02–0.2	4.1	This work

**Table 4:** Determined concentrations of TRZ and corresponding recovery values in pharmaceutical, urine, plasma, and river water samples (ND—not detected).

Sample	Added, µM	Found, µM	Recovery, %
Tablet	0	1.02 ± 0.02	-
	1	2.07 ± 0.14	102.9
	2	3.07 ± 0.06	98.0
Urine diluted 200×	0	ND	-
	1	1.04 ± 0.07	104.1
	2	2.12 ± 0.05	104.2
Plasma diluted 200×	0	ND	-
	1	0.98 ± 0.04	98.4
	2	2.01 ± 0.09	103.2
River water diluted 100×	0	ND	-
	1	1.00 ± 0.12	100.2
	2	1.99 ± 0.27	99.4
River water diluted 200×	0	ND	-
	1	1.02 ± 0.17	102.1
	2	1.99 ± 0.14	97.7

**Table 5:** The efficiency of TRZ extraction into gastric or intestinal juices. The extraction time into gastric juice: 30 or 60 min, and into intestinal juice: 120 min.

Digestive Juice	Tablet	Extraction Efficiency [%]	
Time of extraction [min]		30 min	60 min
Gastric juice	1	31.2 ± 1.8	66.9 ± 1.3
Intestinal juice	1	40.5 ± 5.2	8.02 ± 0.34

## Data Availability

The data presented in this study are available upon request from the corresponding author. The data are not publicly available due to the excessive amount of data in the repository.
